# The release of inhibition model reproduces kinetics and plasticity of neurotransmitter release in central synapses

**DOI:** 10.21203/rs.3.rs-2700789/v1

**Published:** 2023-04-27

**Authors:** Christopher A Norman, Shyam S Krishnakumar, Yulia Timofeeva, Kirill E Volynski

**Affiliations:** 1 Mathematics for Real-World Systems Centre for Doctoral Training, University of Warwick, Coventry, CV4 7AL, UK; 2 University College London Institute of Neurology, University College London, London, WC1N 3BG, UK; 3 Department of Neurology, Yale Nanobiology Institute, Yale University School of Medicine, New Haven, CT 06510, USA; 4 Department of Computer Science, University of Warwick, Coventry, CV4 7AL, UK; 5 Department of Cell Biology, Yale University School of Medicine, New Haven, CT 06510, USA

## Abstract

Calcium-evoked release of neurotransmitters from synaptic vesicles (SVs) is catalysed by SNARE proteins. The predominant view is that, at rest, complete assembly of SNARE complexes is inhibited (‘clamped’) by synaptotagmin and complexin molecules. Calcium binding by synaptotagmins releases this fusion clamp and triggers fast SV exocytosis. However, this model has not been quantitatively tested over physiological timescales. Here we describe an experimentally constrained computational modelling framework to quantitatively assess how the molecular architecture of the fusion clamp affects SV exocytosis. Our results argue that the “release-of-inhibition” model can indeed account for fast calcium-activated SV fusion, and that dual binding of synaptotagmin-1 and synaptotagmin-7 to the same SNARE complex enables synergistic regulation of the kinetics and plasticity of neurotransmitter release. The developed framework provides a powerful and adaptable tool to link the molecular biochemistry of presynaptic proteins to physiological data and efficiently test the plausibility of calcium-activated neurotransmitter release models.

## Introduction

Synaptic release of neurotransmitters forms the basis of information transfer in the brain. It is well established that synaptic vesicle (SV) fusion with the plasma membrane is mediated by SNARE (soluble N-ethylmaleimide–sensitive factor attachment protein receptor) proteins, namely VAMP2 on the SV (v-SNARE) and syntaxin1 and SNAP25 on the plasma membrane (t-SNAREs) in most of central synapses ^1–3^. v- and t-SNAREs can constitutively assemble (or ‘zipper’) into a complex that brings opposing membranes together and provides the energy required for fusion. In addition to synapses, similar SNARE proteins mediate the fusion of virtually all membranous organelles in living cells ^4^.

A distinct property of SV exocytosis is that it is tightly coupled to neuronal activity and controlled by action potential (AP)-evoked increases in presynaptic [*Ca*^2+^]. To achieve this, presynaptic terminals maintain a readily releasable pool (RRP) of vesicles that are docked at the presynaptic active zone (AZ). When an AP reaches the presynaptic terminal, it depolarises the presynaptic membrane and transiently activates voltage-gated Ca^2+^ channels (VGCCs) located in the AZ, resulting in the formation of local Ca^2+^ nano/microdomains near RRP vesicles ([*Ca*^2+^]_*local*_ ~10 – 100 µM). Ca^2+^ ions activate the fast, low-affinity vesicular Ca^2+^ release sensor synaptotagmin 1 (Syt1, or its closely related isoforms Syt2 and Syt9 in different types of synapses), which triggers SV exocytosis and synchronous neurotransmitter release on a millisecond timescale ^3, 5^. VGCCs close within several milliseconds after an AP, resulting in the collapse of Ca^2+^ nano/microdomains. However, the presynaptic Ca^2+^ level remains elevated in the low micromolar range for tens to hundreds of milliseconds. This long-lasting increase in residual [*Ca*^2+^]([*Ca*^2+^]_*residual*_), which is especially prominent during bursts of neuronal activity, triggers delayed asynchronous neurotransmitter release and also contributes to facilitation of synchronous release upon arrival of another AP. This short-term plasticity of vesicular release allows presynaptic terminals to process the neuronal spiking code and provides a basis for synaptic computation and selective information transfer in the brain ^6, 7^. Asynchronous release and synaptic facilitation are, in large part, mediated by the presynaptic membrane-associated high-affinity Ca^2+^-release sensor synaptotagmin 7 (Syt7), which can be activated by ([*Ca*^2+^]_*residual*_)^8–11^. Thus, it is emerging that synaptic release of neurotransmitters is synergistically regulated by low- and high-affinity synaptotagmins acting on the same pool of vesicles or even on the same SNARE complex. How this occurs in molecular terms remains poorly understood.

The current prevailing view is that each RRP vesicle contains several partially assembled SNARE complexes (‘SNAREpins’) that are arrested (‘clamped’) in this state by synaptotagmins and the soluble presynaptic protein complexin. The SNAREpins are thought to be released by Ca^2+^ activation of synaptotagmin molecules and act cooperatively to drive rapid SV exocytosis leading to neurotransmitter release ^12–14^. However, it has not been quantitatively tested whether this ‘release of inhibition’ model can adequately describe the millisecond kinetics of synchronous neurotransmitter release, or if additional mechanisms, such as membrane bending and/or membrane bridging by Syt1 ^15–21^, are also critical.

**Figure 1.**
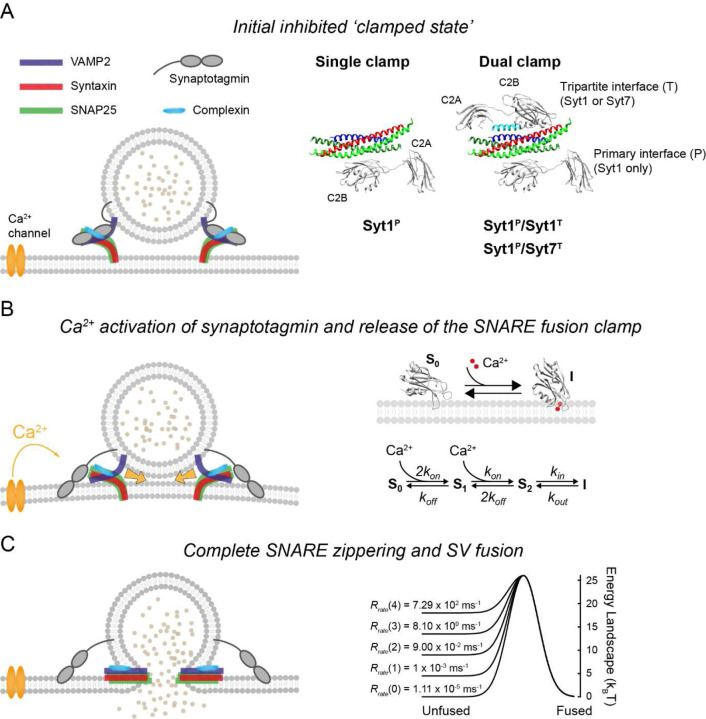
Computational implementation of the release of inhibition model (A). At rest, the full zippering of SNAREs on RRP vesicles is inhibited (‘clamped’) by binding of synaptotagmin and complexin molecules. Based on structural data, three synaptotagmin/SNARE clamp architectures were considered in the model (right). In all cases Syt1 occupies the primary interface. The tripartite interface is either unoccupied (single clamp, Syt1^P^) or occupied (dual clamp) by Syt1 (Syt1^P^/Syt1^T^) or Syt7 (Syt1^P^/Syt7^T^). Crystal structure (PDB ID: 5W5C) ^21^. (B) The binding of two Ca^2+^ ions to a synaptotagmin C2 domain leads to its subsequent membrane insertion (described by the reaction scheme on the right) and release of its SNARE fusion clamp allowing full zippering of SNAREs which provides energy for membrane fusion. (C) The fusion rate is determined by the number of free SNARE complexes that reduce the effective membrane fusion energy barrier, illustrated here as a Gaussian landscape (right). Each SNAREpin was assumed to independently contribute to the lowering of the membrane fusion barrier which is spontaneously overcome at a rate given by the Arrhenius equation (see Methods). Note that only two out of six SNARE complexes are shown in the cartoons on the left that represent a vertical cross-section of the SV.

Recently, considerable progress has been made in understanding the structural and functional organisation of the SNARE pre-fusion complex ^21–25^. It has been shown that a given SNAREpin can simultaneously bind two Syt1 molecules, one at the ‘primary’ interface, independently of complexin, and another at the ‘tripartite’ interface, in conjunction with complexin (Figure 1A) ^21^. Interestingly, the primary site is accessible to only fast, low-affinity Ca^2+^ sensors (Syt1, Syt2 and Syt9), whilst the tripartite site appears to be universally accessible to all synaptotagmin isoforms, including Syt7. Dual synaptotagmin binding at primary and tripartite interfaces has the potential to explain, in molecular terms, how different synaptotagmin isoforms cooperatively regulate neurotransmitter release and short-term plasticity ^10–13^. However, this hypothesis remains to be tested.

One difficulty in addressing these questions is that measuring the spatiotemporal dynamics of Ca^2+^ at the AZ is challenging due to its spatial scale (200 – 600 nm) and the inherently low signal-to-noise ratio of fluorescent Ca^2+^ indicators when imaging with millisecond resolution. Another obstacle is that, at present, it is not possible to directly track the molecular states of different synaptotagmin isoforms on RRP vesicles. Data-constrained realistic computational models of presynaptic terminals are therefore essential tools that can bypass the limitations of experimental approaches. Indeed, we have previously created a set of computational tools to model presynaptic Ca^2+^ dynamics at different types of synapses during physiological patterns of activity ^26–30^.

Here we describe an experimentally constrained computational modelling framework that allows us to model activation of Syt1 and Syt7 by physiologically relevant Ca^2+^ transients that occur at the presynaptic AZ and to test how their activation triggers SV exocytosis for different molecular models of the fusion clamp. We find that release of inhibition is sufficient to explain the millisecond kinetics of AP-evoked SV exocytosis. Our results indicate that, irrespective of the triggering Ca^2+^ signal’s shape, or the nature of the fusion clamp, the majority of synchronous vesicular fusion occurs when 3 SNAREpins are simultaneously free from inhibition. Furthermore, our simulations reveal that the Syt1/SNARE interaction at the primary site alone can account for the millisecond kinetics of AP-evoked synchronous release and that binding of Syt1 or Syt7 to SNARE complexes at the tripartite interface provides an additional level of regulation of vesicular fusion. In particular, dual Syt1/Syt7 binding to the same SNAREpin can explain the role of Syt7 in the regulation of short-term synaptic plasticity and kinetics of vesicular release.

## Results

### Computational implementation of the release of inhibition model.

We assumed that each RRP vesicle was associated with several partially assembled SNAREpins that were clamped in this state by Syt1 and Syt7 isoforms along with complexin (Figure 1A). We considered three synaptotagmin clamp architectures based on the available structural and functional data. In all cases the primary interface was occupied by Syt1. Indeed, the primary interface is selective for Syt1 (and its similar isoforms Syt2 and Syt9) but not for Syt7. It has also been shown that Syt1 can simultaneously interact with the lipid bilayer via PIP2 interaction and SNAREs via the primary interface ^24^. It is thus likely, that Syt1 binding at the primary interface occurs at an early stage of vesicle docking, preceding the SNARE assembly process. In contrast, the tripartite interface is generated only when SNAREs are partially-zippered and can bind complexin. Furthermore, the tripartite interface binding motif is present in both Syt1 and Syt7 ^21^. Therefore, the three synaptotagmin fusion clamp architectures we considered were where the tripartite interface was either unoccupied (single clamp, Syt1^P^) or occupied (dual clamp) either by Syt1 (Syt1^P^/Syt1^T^) or by Syt7 (Syt1^P^/Syt7^T^) (Figure 1A). To model these three limiting cases, we assumed that all SNAREpins on a given RRP vesicle share the same clamp architecture. Biochemical and physiological analyses have shown that a small number of SNAREs are sufficient to achieve fast, Ca^2+^ synchronised neurotransmitter release ^31–33^. Furthermore, recent cryo-electron tomography analysis in cultured hippocampal synapses demonstrated a circular symmetric arrangement of six protein densities at the interface between docked SVs and the presynaptic membrane, each possibly corresponding to a single SNARE-associated exocytic module ^34^. Therefore, we assumed that each RRP vesicle was associated with six SNAREpins.

It is well established that Ca^2+^ binding leads to rapid insertion of the aliphatic loops of synaptotagmin C2 domains into the membrane and that this step is critical for triggering neurotransmitter release ^35, 36^. Indeed, structural and biochemical analyses have indicated that a Ca^2+^-triggered reorientation of Syt1 C2 domains displaces Syt1 from the primary SNARE interface ^24, 37^. Therefore, we assumed that Ca^2+^ binding and subsequent membrane loop insertion of synaptotagmin C2 domains induces removal of the fusion clamp, *i.e.* ‘release of inhibition’ (Figure 1B). Based on the previously established critical roles of the Syt1 C2B domain ^38, 39^ and the Syt7 C2A domain ^8, 9^, for simplicity, we only considered activation of these domains in our model. The C2 domains of Syt1 and Syt7 associated with RRP vesicles are likely to be in close proximity to the membrane ^24, 40, 41^. Hence, we modelled Ca^2+^ triggered loop insertion as a first order reaction described by membrane insertion (*k*_*in*_) and dissociation (*k*_*out*_) rates. Combined with the two-site protein-ligand binding model described by Ca^2+^ binding (*k*_*on*_) and unbinding (*k*_*off*_) rates, the model of Syt1 and Syt7 C2 domain dynamics is described by the kinetic scheme in Figure 1B. This model assumes that Ca^2+^ is not able to dissociate from the C2 domain while it is membrane-inserted, and that reversal of membrane insertion leads to immediate restoration of the SNARE fusion clamp.

Finally, we assumed that the repulsive forces between a SV and the plasma membrane constitute a potential energy barrier, which is lowered by the independent energetic contributions of individual assembled SNARE complexes. SV fusion was triggered when the barrier was overcome by thermal fluctuations at a rate given by the Arrhenius equation (Figure 1C) ^42, 43^. The parameters of the complete Markov model were constrained using the available biochemical and structural data ^13, 21, 24, 40, 41, 44–48^ (see Methods). The fusion dynamics of RRP vesicles in response to diverse [*Ca*^2+^] transients were computed using stochastic Monte Carlo simulations using the Gillespie algorithm ^49^, as detailed in the Methods section.

### The release of inhibition model describes the kinetics of vesicular release at the calyx of Held giant synapse.

The Ca^2+^-activation of SV fusion has been quantitatively described in the calyx of Held - a giant synapse in the auditory brainstem. In these experiments, flash photolysis was used to generate spatially uniform step-like increases of [*Ca*^2+^] within the calyx and the Ca^2+^-evoked vesicular release was monitored using post-synaptic patch-clamp recordings ^50, 51^. The measured kinetics and the [*Ca* ^2+^] dependency of glutamate release at this synapse have been described by several empirical mathematical models ^50–53^. We note that, whilst Syt2 acts as the primary Ca^2+^ sensor for synchronous release in the calyx of Held, its Ca^2+^ and membrane binding properties are very similar to Syt1^46, 54^. Therefore, we used the six-state allosteric model proposed by Lou et al., that closely describes evoked glutamate release over a wide [*Ca*^2+^] range ^52^ as a benchmark for comparison against our simulations (Supplementary Figure 1).

**Figure 2.**
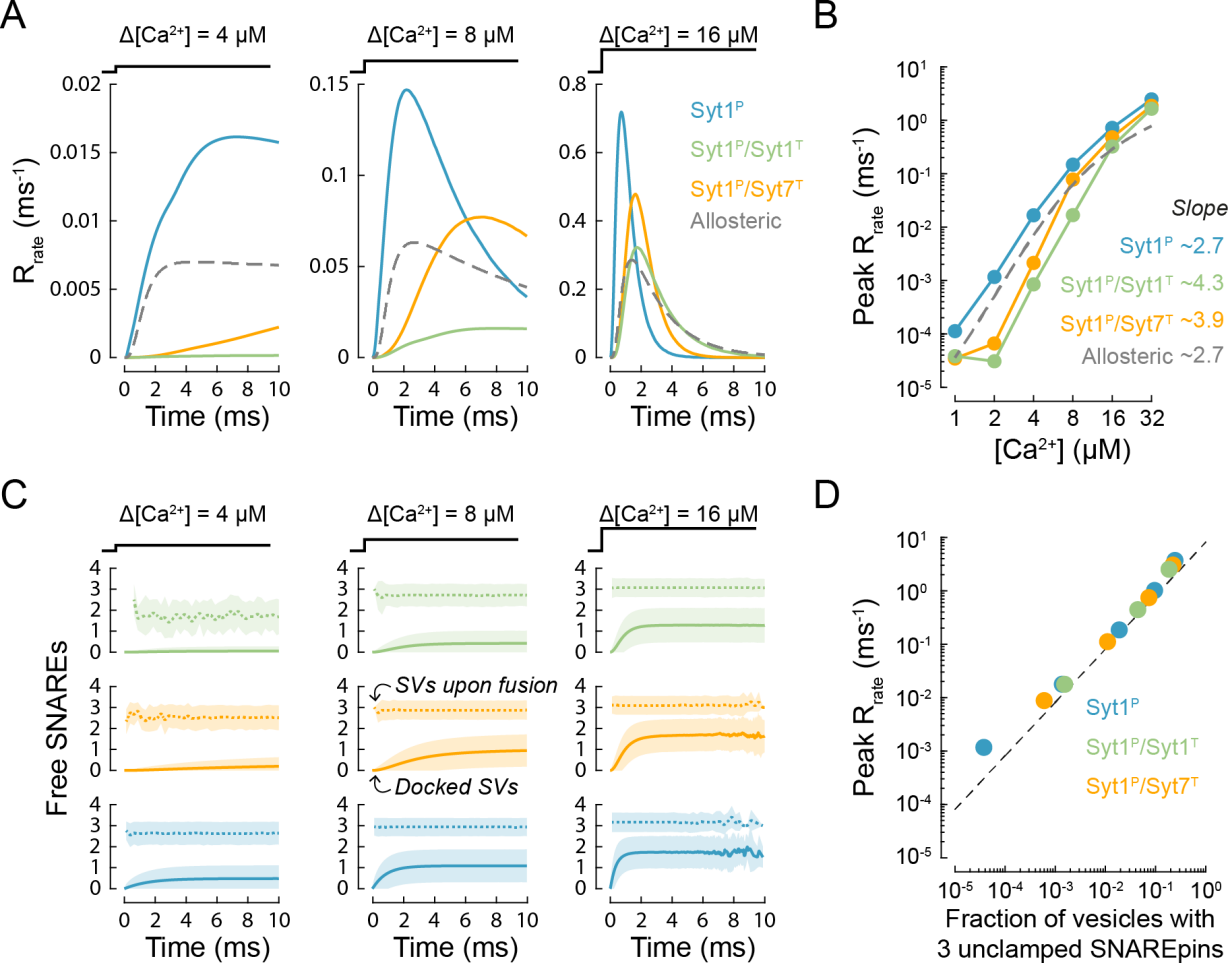
Release of inhibition model describes the kinetics of vesicular release in the calyx of Held. (A) Time-course of vesicular release rate simulated in response to 4 µM, 8 µM, and 16 µM [*Ca*^2+^] steps for the single and dual synaptotagmin/SNARE clamp architectures considered in the model (solid coloured traces) and for the benchmark allosteric model (dashed grey trace) that describes the vesicular release kinetics recorded in the calyx of Held ^52^. (B) Dependency of the peak release rate (achieved within 10 ms) on the amplitude of the [*Ca*^2+^] step. The numbers indicate the slopes corresponding to the exponent of the power relationship between peak vesicular release rate and [*Ca*^2+^] in the range of 4 – 16 µM. (C) Time evolution of the mean number of unclamped SNAREpins (‘Free SNAREs’) on all docked SVs (solid lines), and on SVs at the instance of fusion (dotted lines) in response to [Ca^2+^] step. Shaded areas indicate 1 standard deviation each side of the mean. Each time point includes data from a 0.25 ms bin. The colour code is the same as in (A). (D) The relationship between peak release rate and the fraction of SVs that have 3 unclamped SNARE complexes at the instance of fusion. The dotted line represents an asymptote for the case when fusion may only occur for vesicles that have exactly 3 unclamped SNARE complexes (i.e. the product of the fraction of vesicles with 3 unclamped SNAREs and the Arrhenius rate *R*_*rate*_ (3) = 8.1 ms^−1^ as in Figure 1C). Data points represent mean values taken over a 0.25 ms bin centred on the time of peak release rate. For each [*Ca*^2+^] step and fusion clamp architecture at least *N* = 500,000 stochastic simulations were performed with at least 2,000 vesicular fusion events recorded during the first 10 ms time window. This restricted the normalised root mean squared error in stochastic estimates of the kinetics of SV fusion to less than 1% (See Supplementary Figure 3).

In line with the Ca^2+^ uncaging experiments we simulated vesicular release in response to [*Ca*^2+^] steps in the range of 1 – 32 µM for the three limiting cases of the clamp architecture (Figure 1A). In all three cases, the peak release rates exhibited a power law dependency on the [*Ca*^2+^] step, with exponents between 2.7 and 4.3 (Figure 2A, B). In agreement with the experimental data recorded at the calyx of Held described by the benchmark allosteric model, sub-millisecond fusion rates predicted by the release of inhibition models were apparent for [*Ca*^2+^] steps above 4 µM or 8 µM, depending on the clamp architecture. The release rate was greatest in the case of a single Syt1 clamp at the primary interface. The introduction of an additional Syt1 or Syt7 clamp at the tripartite interface reduced the release rate but enhanced Ca^2+^ cooperativity. The predictions of the allosteric model lie between these limiting clamping cases, demonstrating that the release of inhibition model can indeed explain the experimentally observed kinetics of vesicular release. The model output further suggests that the occupancy of the tripartite interface by different synaptotagmin isoforms could provide an efficient mechanism for the dynamic regulation of Ca^2+^-triggered vesicular release.

To estimate how many SNARE complexes are needed to drive fast synchronous vesicular fusion we monitored the number of unclamped SNAREpins associated with each vesicle prior to fusion. We found that for all clamp architectures, fast fusion (peak release rate above 10^−2^ ms^−1^) required at least 3 uninhibited SNAREpins. This value is consistent with previous experimental and modelling estimates for the number of SNAREs required to mediate synchronous release of neurotransmitters ^31, 32, 43^. Indeed, the apparent rate of synchronous vesicle fusion in our modelling framework is limited by the time taken for 3 SNAREpins to be released from the fusion clamp, which depends on the [*Ca*^2+^] increment and the clamp architecture (Figure 2C). In the case of 3 unclamped SNAREpins the vesicular fusion rate predicted in our model by the Arrhenius equation is 8.1 ms^−1^ (Figure 1C). This value is orders of magnitude greater than the upper limit for how quickly the synaptotagmin clamp can be restored, which is limited by the rate of C2 domain membrane dissociation (*k*_*out*_ = 0.67 ms^−1^ for Syt1 and *k*_*out*_ = 0.02 ms^−1^ for Syt7, see Methods). This implies that once 3 SNAREpins are simultaneously unclamped, vesicle fusion is inevitable. In line with this prediction, the peak release rate was directly proportional to the fraction of vesicles with 3 uninhibited SNAREpins, irrespective of the synaptotagmin clamp architecture (Figure 2D).

### The release of inhibition model recapitulates synchronous release in response to AP-evoked [Ca^2+^] transients at the AZ.

**Figure 3.**
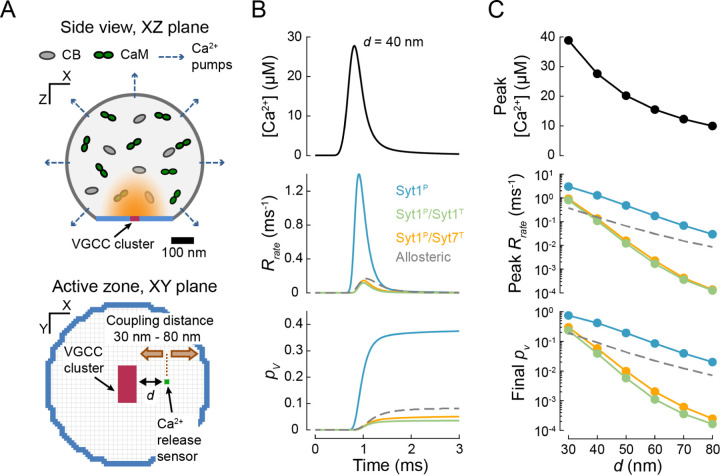
The release of inhibition model recapitulates synchronous release in response to AP-evoked [Ca^2+^] transients in the presynaptic active zone. (A) A cartoon illustrating the model of AP-evoked presynaptic Ca^2+^ dynamics. The presynaptic terminal was modelled as a truncated sphere with a diameter of 0.6 μm with a single AZ containing a 40 nm x 80 nm rectangular cluster of VGCCs. Intraterminal Ca^2+^ dynamics were subject to diffusion, buffering (by calbindin, CB; calmodulin, CaM; and ATP) and extrusion via ion pumps. Illustrations of the volume cross-section (upper) and the AZ plane (lower) are adapted from our previous work ^29^. (B) Time course of AP-evoked [*Ca*^2+^](*t*) at an AZ point 40 nm from the VGCC cluster (top) and the corresponding vesicular release rates (middle) and the cumulative vesicular release probability, *p*_*v*_ (bottom) for the three clamp architectures (solid coloured lines) and the benchmark allosteric model (dashed grey line). (C) Dependences of peak [*Ca*^2+^] (top), peak vesicular release rate (middle) and ‘final’ *p*_*v*_ calculated 2 ms after an AP (bottom) on the coupling distance *d* between the docked vesicle and the VGCC cluster. Colour codes as in (B). Data points represent mean values. For each [*Ca*^2+^](*t*) transient and fusion clamp architecture at least *N* = 500,000 stochastic simulations were performed with at least 2,000 vesicular fusion events recorded during the first 3 ms time window.

We next examined whether the release of inhibition model could replicate the vesicular release dynamics observed at presynaptic terminals in response to AP stimulation. AP-evoked Ca^2+^ dynamics at vesicular release sites have not been directly measured, largely because of the small size of the AZ and the high speed of Ca^2+^ kinetics. Therefore, we and others have developed three-dimensional, experimentally constrained models of Ca^2+^ influx, diffusion, buffering and extrusion which allow one to approximate the [*Ca*^2+^] transients near release-ready vesicles at different types of synapses ^26–30, 55, 56^. A critical parameter that determines [*Ca*^2+^]_*local*_ at a given release site is its distance to the nearest VGCC cluster, *i.e.* the coupling distance, *d*. As in our previous work ^29^, we considered a simplified model of a small excitatory presynaptic terminal that contains a single active zone with a cluster of VGCCs at the centre, and computed AP-evoked [*Ca*^2+^]_*local*_ for coupling distances ranging between 30 – 80 nm, which is characteristic for small central glutamatergic synapses (Figure 3A and Methods). We then extracted [*Ca*^2+^]_*local*_ at different coupling distances and used these as model inputs to simulate vesicular fusion (Figure 3B, C).

In line with the analysis of step [*Ca*^2+^] increments (Figure 2A, B), the model-predicted vesicular release kinetics in response to AP-evoked Ca^2+^ influx depended on the architecture of the synaptotagmin clamp (Figure 3). For all coupling distances tested, the vesicular release probability was highest when the tripartite interface was unoccupied (single Syt1^P^ clamp). Adding either a second Syt1 or Syt7 clamp at the tripartite interface reduced the vesicular release by similar amounts. The amplitude of AP-evoked [*Ca*^2+^] transients decreased with increasing coupling distance, which resulted in a corresponding reduction of both the peak release rate and the overall release probability (Figure 3C). The decrease was steeper when the tripartite site was occupied by either Syt1 or Syt7 due to increased Ca^2+^ cooperativity. Notably, the predictions of the benchmark allosteric model were within the range of the predictions of the three limiting cases of clamping architectures. These results further demonstrate that the release of inhibition model can reproduce the [*Ca*^2+^] dependency and fast kinetics of AP-evoked synchronous vesicular release.

### The release of inhibition model reproduces Syt7-dependent short-term facilitation.

**Figure 4.**
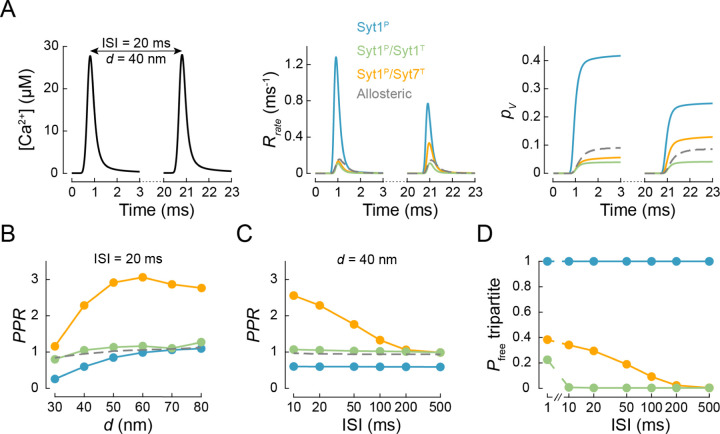
The release of inhibition model reproduces Syt7-dependent short-term facilitation (A) Modelling of vesicular release in response to paired-pulse stimulation. Left, VCell computed Ca^2+^ dynamics at a vesicular release site located at *d* = 40 nm from the VGCC cluster (see Figure 3A) in response to a pair of APs separated by 20 ms (ISI, interstimulus interval). Time course of the corresponding vesicular release rates (middle) and the cumulative vesicular release probability (*p*_*v*_) (right) for the three fusion clamp architectures (solid coloured lines) and the benchmark allosteric model (dashed grey line). (B, C) Dependency of the paired-pulse ratio (*PPR* = *p*_*v*_ (2) / *p*_*v*_ (1), where *p*_*v*_ (1) and *p*_*v*_ (2) are the vesicular release probabilities at the 1^st^ and the 2^nd^ AP respectively), on the coupling distance (B) and the interstimulus interval (C) for different clamp architectures. Note that facilitation of vesicular release was only observed when Syt7 was present (Syt1^P^/Syt7^T^). Colour codes as in (A). (D) Probability that the tripartite interface remains unoccupied (*P*_*free*_ tripartite) at the time of arrival of the 2^nd^ AP. Due to the faster membrane dissociation rate of Syt1 (*k*_*out*_ = 0.67 ms^−1^), the model predicts restoration of the Syt1 clamp within 10 ms after the 1^st^ AP. In contrast, Syt7 exhibits slower membrane dissociation (*k*_*out*_ = 0.02 ms^−1^) which leads to a delayed restoration of the fusion clamp and results in the facilitation of vesicular release at the 2^nd^ AP. Colour codes as in (A). Data points represent mean values. For each [*Ca*^2+^](*t*) transient and fusion clamp architecture at least *N* = 500,000 stochastic simulations were performed with at least 2,000 vesicular fusion events recorded during the first for each AP.

Short-term plasticity of synaptic neurotransmitter release is commonly assessed by measuring vesicular release in response to pairs of APs. We therefore tested how the molecular architecture of the synaptotagmin clamp shapes vesicular release in response to paired-pulse stimulation (Figure 4). Using the VCell model described in Figure 3 we computed [*Ca*^2+^] responses to different inter-stimulus intervals (10 to 500 ms) and coupling distances (30 to 80 nm). We next simulated vesicular release for different synaptotagmin clamp architectures in response to the obtained Ca^2+^ dynamics and calculated the paired-pulse ratio *PPR* = *p*_*v*_ (2) / *p*_*v*_ (1) (where *p*_*v*_ (1) and *p*_*v*_ (2) are vesicular release probabilities at the 1^st^ and the 2^nd^ AP respectively).

In the case of the benchmark allosteric model, vesicular release was similar in response to the 1^st^ and 2^nd^ AP (Figure 4A–C). This was expected since [*Ca*^2+^] transients at vesicular release sites were comparable for the 1^st^ and 2^nd^ AP (Figure 4A). In the case of the single Syt1 clamp at the primary interface (Syt1^P^), we observed depression of vesicular release in response to the 2^nd^ AP, which was most prominent at short coupling distances where [*Ca*^2+^]_*local*_ transients had the greatest amplitude. The depression can be explained by depletion of the RRP vesicles due to the absence of vesicle replenishment in these simulations and the relatively high release probability at the 1^st^ AP (Figure 3C and Figure 4A). In contrast, in the case of the dual Syt1 clamp (Syt1^P^/Syt1^T^) the release probability at both the 1^st^ and the 2^nd^ APs was low, and the *PPR* was close to 1 for the whole range of coupling distances and inter-spike intervals tested. Strikingly, inclusion of Syt7 at the tripartite interface (Syt1^P^/Syt7^T^) led to a facilitation of vesicular release at the 2^nd^ AP (Figure 4A–C). The increase of *PPR* was most noticeable (2- to 3-fold) at shorter inter-stimulus intervals (10 – 50 milliseconds) and longer coupling distances (> 40 nm).

The observed facilitation can be explained by the slower membrane dissociation kinetics of Syt7 relative to Syt1^45^. The 1^st^ AP induces the insertion of Syt1 and Syt7 C2 domains into the membrane, which leads to release of the fusion clamp at a fraction of SNAREpins. If fusion of a given vesicle was not successfully induced during the 1^st^ AP, then the clamp on its SNAREpins would be restored more slowly by Syt7 than by Syt1, because Syt7 stays longer in the membrane (*k*_*out*_ = 0.02 ms^−1^ for Syt7 and 0.67 ms^−1^ for Syt1). This means that at the time of arrival of the 2^nd^ AP these unfused vesicles are expected to have up to 40% of Syt7 clamps already released (Figure 4D), conferring an increase in the probability of SV fusion, *p*_*v*_(2).

The observed short-term facilitation (STF) mediated by Syt7 diminished as the inter-pulse interval increased, due to progressive restoration of the Syt7 clamp, and disappeared within 500 ms, consistent with experimental data ^11, 57^. Indeed, the dependency of *PPR* on the inter-stimulus interval for a given coupling distance aligned well with that of the average number of free Syt7 clamps immediately before onset of the 2^nd^ AP (Figure 4C, D). In contrast, the dependency of Syt7-mediated STF on coupling distance was non-monotonic, with *PPR* reaching maximal values at coupling distances between 50 – 60 nm (Figure 4B). This was due to the competing effects of increased vesicle depletion after the 1^st^ AP at short coupling distances versus decreased removal of Syt7 clamps on unfused vesicles at longer coupling distances.

### Mixed single and dual fusion clamp model can recapitulate vesicular release properties at plastic synapses.

We next assessed whether the release of inhibition model can explain the complex patterns of vesicular release observed in response to bursts of neuronal activity. To test this, we chose the mossy fibre bouton (MFB) – CA3 pyramidal neuron synapse in the hippocampus as a model environment. This synapse, which is also called a ‘detonator synapse’, has a very low initial release probability (*p*_*v*_ for individual RRP vesicles is in the range of 0.01 – 0.03) and shows strong STF of synchronous release (up to 10-fold) and prominent asynchronous release after high frequency bursts of activity ^58, 59^. STF in MFBs is mediated by at least two different mechanisms: (i) progressive increase of peak [*Ca*^2+^]_*local*_ at vesicular release sites due to Ca^2+^ buffer saturation and (ii) activation of Syt7 due to increase of [*Ca*^2+^]_*residual*_^8, 28^.

**Figure 5.**
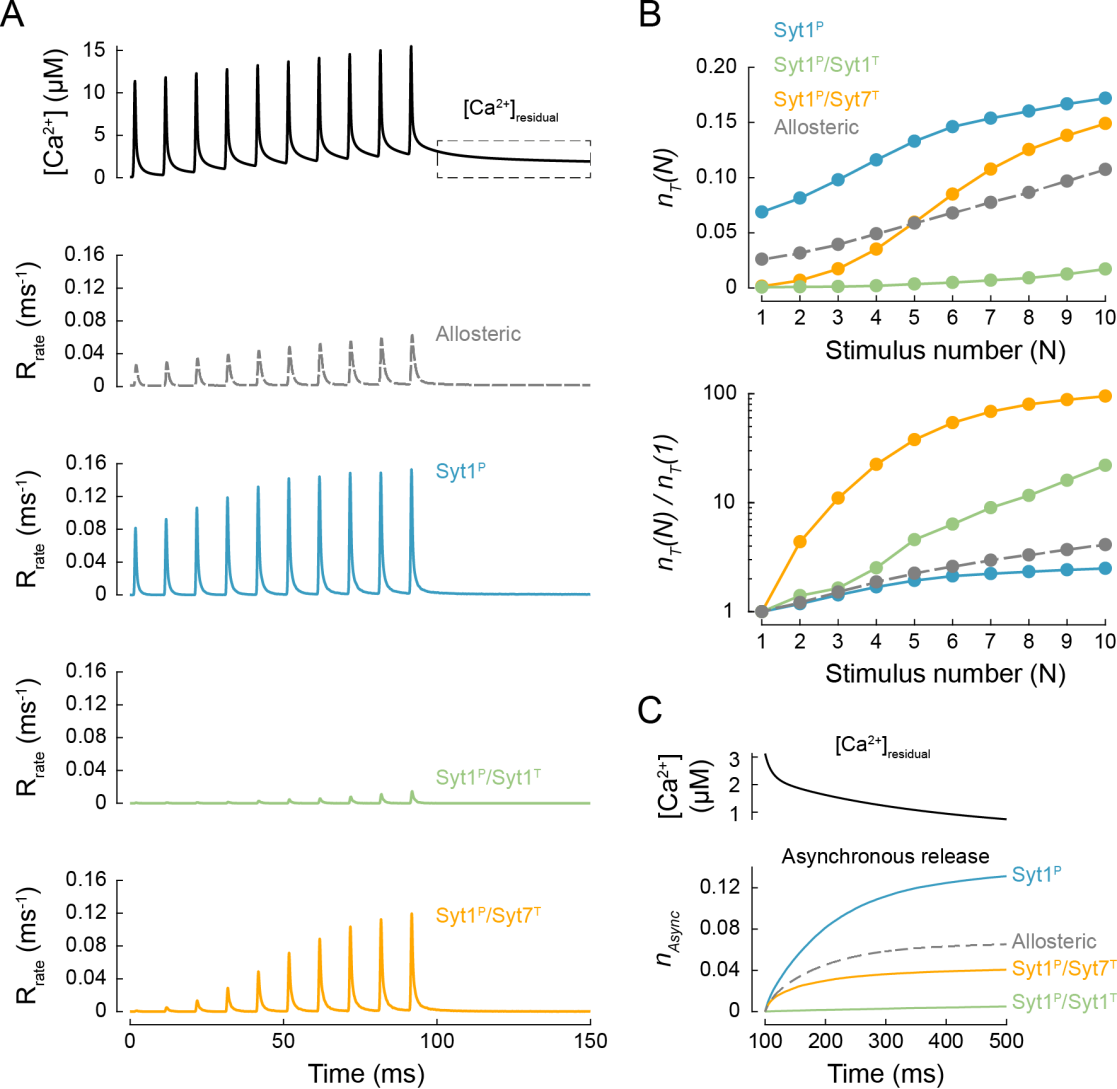
Modelling vesicular release for single and dual clamp models in response to bursts of action potentials in MFB terminals. (A) Top, VCell computed [*Ca*^2+^](*t*) transient approximating Ca^2+^ dynamics at vesicular release sites in MFB terminals in response to 10 × 100 Hz AP train (see ref.^28^). Bottom, time course of corresponding vesicular release rates for the benchmark allosteric model and the three fusion clamp architectures. (B) Top, expected numbers of vesicles exocytosed at a single release site, *n*_*T*_ (*N*), for each AP of the train, *N*. Bottom, paired-pulse ratio: release efficacy at each AP normalised to release efficacy at the first AP, *n*_*T*_ (*N*) / *n*_*T*_ (1). (C) Top, residual [*Ca*^2+^]_*residual*_ after the 10 × 100 Hz AP train, corresponding to the dashed box in (A). Bottom, corresponding cumulative asynchronous releases per release site, *n*_*Async*_, for the three fusion clamp architectures and the benchmark allosteric model. Data points represent mean values. For each [*Ca*^2+^](*t*) transient and fusion clamp architecture at least *N* = 100,000 stochastic simulations were performed with at least 6,000 vesicular fusion events recorded during the AP train.

We previously constrained Ca^2+^ dynamics at MFB release sites during high-frequency bursts of APs using a three-dimensional VCell model ^27, 28^. Here we used the previously estimated [*Ca*^2+^]_*local*_ transient at the AZ in response to a 100 Hz train of 10 Aps as a model input, and simulated vesicular release responses for the three fusion clamp architectures and the benchmark allosteric model (Figure 5). In this set of simulations, we also implemented vesicle replenishment after a fixed refractory time of 2.5 ms^−1^ with a rate of *k*_*rep*_ = 0.02 ms^−1^ (as estimated in ref. ^28^). Therefore we used the release efficacy, *n*_*T*_, defined as the expected number of vesicles exocytosed at a single release site, intead of *p*_*v*_ to compare vesicular release among different models.

The release efficacy after the first AP predicted by the benchmark model was in the physiological range, *n*_*T*_ (1) = 0.026. The allosteric model also replicated short-term facilitation, with synchronous release at the 10^th^ stimulus approximately 4-fold higher than at the 1^st^ stimulus, *n*_*T*_ (10)/*n*_*T*_ (1) = 4.1. The degree of facilitation was somewhat lower than the experimentally observed value: *n*_*T*_ (10)/*n*_*T*_ (1) in the range of 5 to 10 ^8, 28^. Indeed, by its design the allosteric model accounts for facilitation triggered by increases in peak [*Ca*^2+^]_*local*_ vesicular release sites (Figure 5A), but not the effect of Syt7 activation resulting from a build-up of [*Ca*^2+^]_*residual*_.

In the case of a single Syt1 clamp (Syt1^P^) the initial release efficacy was several-fold higher than in the case of the benchmark allosteric model, *n*_*T*_ (1) = 0.07, and the model predicted only modest facilitation with *n*_*T*_ (10)/*n*_*T*_ (1) = 2.5. Addition of a second Syt1 or Syt7 clamp essentially eliminated vesicular fusion at the 1^st^ AP with *n*_*T*_ (1) = 0.0008 for Syt1^P^/Syt1^T^ and 0.0015 for Syt1^P^/Syt7^T^. Consequently, both dual clamp models showed very strong facilitation, *n*_*T*_ (10)/*n*_*T*_ (1) = 20 for Syt1 ^P^/Syt1^T^ and 100 for Syt1^P^/Syt7^T^ (Figure 5 A–C).

**Figure 6.**
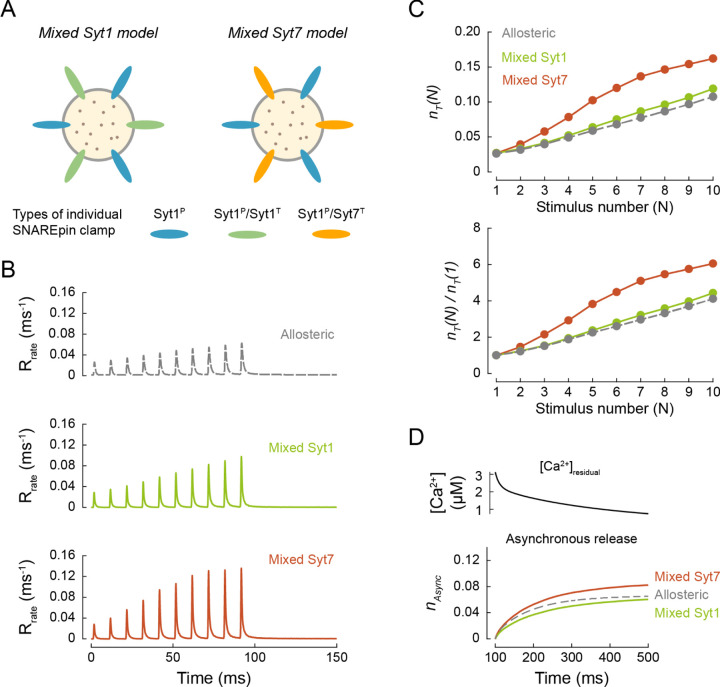
Mixed single and dual clamp model can describe the kinetics and plasticity of release in MFBs. (A) We considered two partial dual clamp models where on average 3 out of 6 SNAREpins had a single Syt1^P^ clamp, and the other 3 had dual Syt1^P^/Syt1^T^ or Syt1^P^/Syt7^T^ clamps. (B) Time course of vesicular release rates for the benchmark allosteric model and the Mixed Syt1 and Mixed Syt7 models in response to the same [*Ca*^2+^](*t*) transient as in Figure 5A. (C) Top, expected numbers of vesicles exocytosed at a single release site, *n*_*T*_ (*N*), for each AP of the train, *N*. Bottom, paired-pulse ratio: release efficacy at each AP normalised to release efficacy at the first AP, *n*_*T*_ (*N*) / *n*_*T*_ (1). (D) Top, residual [*Ca*^2+^]_*residual*_ after the 10 × 100 Hz AP train, corresponding to the dashed box in Figure 5A. Bottom, corresponding cumulative asynchronous releases per release site, *n*_*Async*_, for the two mixed clamp architectures and the benchmark allosteric model. Data points represent mean values. For each [*Ca*^2+^](*t*) transient and fusion clamp architecture at least *N* = 150,000 stochastic simulations were performed with at least 100,000 vesicular fusion events recorded during the AP train.

Considering that neither the single nor the dual clamp architecture could fully recapitulate all the different facets of vesicular release at MFBs, we tested if a mixture of single and dual synaptotagmin clamps could better describe the physiological data. We considered two models. In both cases, on average, half of the SNAREpins were clamped by Syt1 at the primary interface only, whilst the remaining SNAREpins had a dual clamp arrangement with either Syt1 or Syt7 at the tripartite interface (Mixed Syt1 model and Mixed Syt7 model respectively, Figure 6A). In comparison to the full dual clamp models considered above, partial removal of the clamp from the tripartite interface increased the initial release efficacy for individual RRP vesicles to the physiological level, *n*_*T*_(1) = 0.014 for the Mixed Syt1 model and 0.015 for the Mixed Syt7 model. Both mixed models showed prominent STF which was stronger for the Mixed Syt7 model, *n*_*T*_(10)/*n*_*T*_(1) = 10.6, than the Mixed Syt1 model, *n*_*T*_(10)/*n*_*T*_(1) = 6.1. This is because the progressive increase of peak [*Ca*^2+^]_*local*_ is the sole determinant of facilitation in the case of Mixed Syt1 model. In contrast, Syt7 is activated by the elevation of [*Ca*^2+^]_*residual*_ which leads to progressive removal of Syt7 clamps during the burst in the case of the Mixed Syt7 model (see also Figure 4D). This result is consistent with the decreased STF observed in MFB synapses of Syt7 knockout versus wild type mice ^8^.

We also tracked the asynchronous release component triggered by elevated [*Ca*^2+^]_*residual*_ after the AP burst (Figure 5C and Figure 6D). As expected, we found that the rate of asynchronous release depended on the fusion clamp architecture. The asynchronous release was prominent in the single Syt1^P^, dual Syt1^P^/Syt7^T^ and Mixed Syt1 and Syt7 models, whereas no asynchronous release was observed in the case of the dual Syt1^P^/Syt1^T^ clamp model.

These results further illustrate that the balance between single and dual synaptotagmin clamp arrangements could provide a mechanism for the regulation of both short-term synaptic plasticity and the kinetics of vesicular release.

## Discussion

Our computational analysis shows that the release of inhibition model, *i.e.* Ca^2+^-triggered removal of the SNARE fusion clamp, can indeed explain the kinetics of evoked neurotransmitter release. Our simulations further show that fast SV fusion requires simultaneous release of inhibition of at least 3 SNAREpins. Thus, the kinetics of vesicular fusion depend on how rapidly this state is reached, which is in turn determined by the shape and the amplitude of the [*Ca* ^2+^] transient at the AZ and by the architecture of the fusion clamp.

Based on the available structural data ^21^ we considered three possible limiting cases of the fusion clamp architecture: with a single Syt1 clamp at the primary interface (Syt1^P^) and dual Syt1 and/or Syt7 clamps at primary and tripartite interfaces (Syt1^P^/Syt1^T^ and Syt1^P^/Syt7^T^). Our analysis shows that the release of a single or dual synaptotagmin clamp can account for sub-millisecond kinetics of Ca^2+^ triggered neurotransmitter exocytosis. Furthermore, the dual binding Syt1^P^/Syt7^T^ arrangement also reproduced STF of SV exocytosis in response to pairs or burst of APs. This result shows that the release of inhibition model also provides a mechanism by which Syt7 can regulate short-term plasticity.

The functional importance of the primary interface has been well established, both in live synapses and under reconstitution conditions ^22, 23, 33, 60^. In contrast, the relevance of the tripartite interface remains unclear because the interaction of SNAREs/synaptotagmins/complexin at this site cannot be measured biochemically and is thus expected to be very weak ^21, 33, 37, 61, 62^. However, considering the high local concentration of the vesicular release machinery components at RRP vesicles, it is reasonable to expect that the tripartite interface can be at least partially occupied under physiological conditions. In fact, our simulation argues that the weak interaction at this site may play an important functional role. We find that under all conditions tested the release rates predicted by the single Syt1^P^ clamp model were higher than those predicted by the benchmark empirical allosteric model. In contrast, the vesicular fusion rates predicted by both Syt1^P^/Syt1^T^ and Syt1^P^/Syt7^T^ dual clamp models were below physiological levels. Thus, the dynamic occupancy of the tripartite interface by either Syt1 or Syt7 could provide direct control of kinetics and plasticity of neurotransmitter release by changing the strength and Ca^2+^-activation properties of the SV fusion clamp. Indeed, we find that a mixed model combining both single and dual clamp architectures can closely describe the release kinetics and short-term plasticity in response to trains of APs in hippocampal MFB terminals.

While our parsimonious model describes the key molecular elements of Ca^2+^ activation of synaptotagmin and removal of the fusion clamp, it has several simplifications. We assumed that release of the fusion clamp occurs simultaneously with Ca^2+^-triggered membrane insertion of the C2 domain. This is likely the case for the primary interface ^24^, but the kinetics of synaptotagmin/complexin/SNARE interactions at the tripartite interface are not currently known. Similarly, we assumed that restoration of the clamp occurs instantaneously after reversal of synaptotagmin membrane insertion. Thus, the current model provides an upper estimate for the magnitude of synchronous release and lower estimates for asynchronous release and short-term facilitation. Furthermore, in addition to synaptotagmins and canonical SNAREs considered in our model, different modes of Ca^2+^-evoked release might utilize vesicles with distinct compositions of SNAREs and Ca^2+^ sensors (*e.g.* VAMP4 ^63^, VAMP7 ^64^ and Doc2 ^65^).

For simplicity, we only modelled the activation of Syt1 C2B and Syt7 C2A domains. Considering that the C2A and C2B domains act synergistically ^66^, inclusion of the second C2 domain in the model will increase the overall Ca^2+^ / membrane affinity of synaptotagmin molecules and the Ca^2+^ cooperativity. At present, the binding modalities of Syt7 to the SNARE complex remain unknown; thus, we modelled Syt7 binding to SNAREs via the tripartite interface based on structural homology ^21^. However, it is worth noting that the output for the Syt1/Syt7 dual clamp model will be the same even if Syt7 binds at a different site. Finally, the current model does not account for possible roles of Syt1 oligomerisation ^67, 68^, mechanical coupling among different SNAREpins ^43^, and membrane remodelling activities of Syt1 that occur after the release of inhibition ^15–21^.

Nevertheless, the model can be easily modified to implement any of the above mechanisms as well as other fusion clamp architectures. Therefore, the developed modelling framework provides a powerful and adaptable tool to complement experimental work and gain insights into how the presynaptic vesicular release machinery decodes Ca^2+^ signals and translates them into complex patterns of neurotransmitter release.

## Materials and Methods

### Release of inhibition model parameters.

We considered that within the physiological range of [*Ca*^2+^] observed at the presynaptic AZ (~ 50 nM – 200 µM) Syt1 and Syt7 C2 domains can bind two Ca^2+^ ions independently, with similar intrinsic affinities *K*_*d*_ ~ 150 µM ^44, 45^. It has been shown that the rate of Ca^2+^ binding by synaptotagmin C2 domains is limited by diffusion (~ 0.1 – 10 µM^−1^ ms^−1^) ^48^. Therefore, we assumed *k*_*on*_ = 1 µM^−1^ ms^−1^. Because of the symmetry between the free and fully Ca^2+^-bound states (*S*_0_ and *S*_2_ respectively in the kinetic scheme Figure 1B), the dissociation rate constant can be expressed as *k*_*off*_ = *k*_*on*_ · *K*_*D*_, which yielded *k*_*off*_ = 150 ms^−1^ based on the intrinsic Ca^2+^ affinity *K*_*d*_ = 150 µM. The characteristic time for synaptotagmin C2 domain rotation and membrane insertion has previously been estimated as ~10 µs ^14^, which corresponds to a rate of *k*_*in*_ = 100 ms^−1^. Exponential rate constants describing the apparent rates of C2 domain dissociation from lipid membranes (*k*_*diss*_) were previously measured in the presence of EGTA using stopped-flow experiments and reported to be in the ranges of 0.38 – 0.7 ms^−1^ for Syt1, and 0.008 – 0.02 ms^−1^ for Syt7 ^46–48^. We used representative values of *k*_*diss*_ = 0.5 ms^−1^ for Syt1 and *k*_*diss*_ = 0.015 ms^−1^ for Syt7. We estimated the actual rates at which C2 domain aliphatic loops dissociate from the membrane to be *k*_*out*_ = 0.67 ms^−1^ for Syt1 and *k*_*out*_ = 0.02 ms^−1^ for Syt7 by simulating the stopped-flow experiments with our kinetic model (Supplementary Figure 2).

The rate of SNARE-mediated SV fusion was determined by assuming that the repulsive forces between a docked SV and the plasma membrane amount to an energy barrier of *E*_0_ ≈ 26 k_B_T ^42^. Overcoming this barrier requires bringing the SV to within around 1–2 nm of the plasma membrane such that membrane fusion is spontaneously induced ^14^. The full assembly of a single SNARE complex from a half-zippered state has been estimated to provide ∆*E* ≈ 4.5 k_B_T of work towards overcoming the resting energy barrier ^43^. We assumed that ∆*E* is made immediately available to the vesicle in the form of potential energy when a SNAREpin is freed from its synaptotagmin clamp, effectively lowering the energy barrier to membrane fusion. Thus, with *n* uninhibited SNAREpins, the barrier to fusion has a height of *E*_0_ – *n*∆*E* and is spontaneously overcome through thermal fluctuations at a rate given by the Arrhenius equation $R_{rate}(n)=A\cdot \exp\left(-\frac{E_{0}-n\Delta E}{k_{B}T}\right)$ (Figure 1C). We estimated the pre-factor *A* = 2.17×10^9^ s^−1^, considering that a single SNARE complex can mediate fusion *in vitro* on a time scale of 1 s ^14, 43^.

### Implementation of Stochastic Simulations.

All simulations and analysis were carried out in MATLAB 2020b, The MathWorks, Inc. The predicted responses of the release of inhibition model and the benchmark allosteric model to input [*Ca*^2+^](*t*) traces were generated by Monte Carlo estimation from multiple stochastic simulations. Specifically, we used the direct Gillespie algorithm ^49^ which proceeds by iteratively generating a randomised time at which the system next changes its state and then randomly selecting the identity of the new state. The Ca^2+^ binding and SV fusion dynamics of the allosteric model were described by a six-state kinetic scheme with a single occupied state which is updated at each step of the algorithm. The release of inhibition models consisted of either six (in the case of Syt1^P^) or twelve (in the cases of Syt1^P^/Syt1^T^ and Syt1^P^/Syt7^T^) four-state kinetic schemes (Figure 1B), one for each Syt C2 domain. Rather than updating a unique state in the resultant macroscopic Markov chain which had either 4^6^ or 4^12^ states, we monitored each synaptotagmin C2 domain concurrently and updated one of their states according to the algorithm. We assumed that at the start of each simulation both the Ca^2+^ sensor in the benchmark allosteric model and all SNARE-associated synaptotagmin C2 domains in the release of inhibition models were in the Ca^2+^ unbound state.

In simulations shown in Figures 2, 3 and 4 we did not include the mechanism for vesicle replenishment and individual stochastic simulations terminated when vesicle fusion occurred. In simulations describing vesicular release in response to a 10 × 100 Hz AP train we included a mechanism for SV replenishment. Upon vesicle fusion the release site remained unoccupied for a fixed refractory time of 2.5 ms^−1^ after which a SV was replenished in the initial state with the rate of *k*_*rep*_ = 0.02 ms^−1^, as was estimated in our previous work ^28^.

For each scenario the collection of stochastic simulations yielded a set of times at which SV fusion occurred. We used the cumulative count of these vesicle fusion times, normalised to the total number of stochastic simulations, as an estimate for the expected cumulative number of vesicles exocytosed at a single release site by time *t*(*n*_*T*_(*t*)). In the absence of vesicle replenishment *n*_*T*_(*t*) corresponds to the cumulative vesicular release probability *pv*(*t*). In production, *n*_*T*_(*t*) was calculated by gathering release event times into a histogram with an adaptive bin width to capture the features of release kinetics at different temporal scales. The release rate was estimated as $\frac{dn_{T}\left(t\right)}{dt}$ with a moving average smoothing applied to limit the sensitivity of peaks to stochastic variation.

In order to assess the accuracy of these release estimates generated using the Monte Carlo approach we compared the *n*_*T*_(*t*) values obtained from stochastic simulations of the benchmark allosteric model to the *n*_*T*_(*t*) values obtained by numerical solution of the allosteric model’s differential master equations. Given a set of *N* release events, we found that the normalised root mean squared error in Monte Carlo predictions converged at a rate of approximately $\frac{1}{\sqrt{N}}$ (Supplementary Figure 3). For each scenario considered with both the allosteric and release of inhibition models, by default, stochastic simulations were set to continue until 10^5^ release events had been recorded. This corresponded to an average error of less than 0.1%. However, due to resource constraints and differences in computational demand between scenarios, this was not always achieved, and the total number of simulated release events varied. Across all scenarios the minimum number of release events recorded was 2,250 (Syt1^P^/Syt1^T^ at a 2 µM step), corresponding to an average error of less than 1%, making this the expected upper limit of prediction errors due to stochastic variation.

### Simulation of [Ca^2+^] transients within a presynaptic terminal.

Three-dimensional modelling of AP-evoked presynaptic Ca^2+^ influx, buffering, diffusion and extrusion was performed in the Virtual Cell (VCell) simulation environment (vcell.org) using the fully implicit adaptive time-step finite-volume method on a 10-nm meshed geometry as described in detail in our previous studies. Specifically, [*Ca*^2+^](*t*) transients approximating Ca^2+^ dynamics at the AZ of small excitatory boutons (Figures 3 and 4) and MFB terminals (Figures 5 and 6) were computed using the VCell models described in ref. ^29^ and ref. ^28^ respectively. The properties of endogenous Ca^2+^ buffers used in the model are shown in Supplementary Table 1.

## Supplementary Material

Supplement 1

## References

[1] Sollner T. SNAP receptors implicated in vesicle targeting and fusion. Nature 362, 318–324 (1993). 8455717 10.1038/362318a0

[2] Weber T. SNAREpins: minimal machinery for membrane fusion. Cell 92, 759–772 (1998). 9529252 10.1016/s0092-8674(00)81404-x

[3] Sudhof T.C. Neurotransmitter release: the last millisecond in the life of a synaptic vesicle. Neuron. 80, 675–690 (2013). 24183019 10.1016/j.neuron.2013.10.022 PMC3866025

[4] Sudhof T.C. & Rothman J.E. Membrane fusion: grappling with SNARE and SM proteins. Science. 323, 474–477 (2009). 19164740 10.1126/science.1161748 PMC3736821

[5] Kaeser P.S. & Regehr W.G. Molecular mechanisms for synchronous, asynchronous, and spontaneous neurotransmitter release. Annu. Rev. Physiol. 76:333–63. doi: 10.1146/annurev-physiol-021113-170338. Epub@2013 Nov 21., 333–363 (2014). 24274737 PMC4503208

[6] Reyes A. Target-cell-specific facilitation and depression in neocortical circuits. Nat. Neurosci. 1, 279–285 (1998). 10195160 10.1038/1092

[7] Abbott L.F. & Regehr W.G. Synaptic computation. Nature. 431, 796–803 (2004). 15483601 10.1038/nature03010

[8] Jackman S.L., Turecek J., Belinsky J.E., & Regehr W.G. The calcium sensor synaptotagmin 7 is required for synaptic facilitation. Nature. 529, 88–91 (2016). 26738595 10.1038/nature16507 PMC4729191

[9] Bacaj T. Synaptotagmin-1 and synaptotagmin-7 trigger synchronous and asynchronous phases of neurotransmitter release. Neuron 80, 947–959 (2013). 24267651 10.1016/j.neuron.2013.10.026 PMC3888870

[10] Huson V. & Regehr W.G. Diverse roles of Synaptotagmin-7 in regulating vesicle fusion. Curr. Opin. Neurobiol. 63:42–52. doi: 10.1016/j.conb.2020.02.006., 42–52 (2020). 32278209 PMC10305731

[11] Jackman S.L. & Regehr W.G. The Mechanisms and Functions of Synaptic Facilitation. Neuron 94, 447–464 (2017). 28472650 10.1016/j.neuron.2017.02.047 PMC5865607

[12] Brunger A.T., Leitz J., Zhou Q., Choi U.B., & Lai Y. Ca(2+)-Triggered Synaptic Vesicle Fusion Initiated by Release of Inhibition. Trends Cell Biol. 10 (2018). 10.1016/j.tcb.2018.03.004 PMC6056330 29706534

[13] Volynski K.E. & Krishnakumar S.S. Synergistic control of neurotransmitter release by different members of the synaptotagmin family. Curr. Opin. Neurobiol. 51, 154–162 (2018). 29886350 10.1016/j.conb.2018.05.006

[14] Rothman J.E., Krishnakumar S.S., Grushin K., & Pincet F. Hypothesis - buttressed rings assemble, clamp, and release SNAREpins for synaptic transmission. FEBS Lett. 591, 3459–3480 (2017). 28983915 10.1002/1873-3468.12874 PMC5698743

[15] Hui E., Johnson C.P., Yao J., Dunning F.M., & Chapman E.R. Synaptotagmin-mediated bending of the target membrane is a critical step in Ca(2+)-regulated fusion. Cell. 138, 709–721 (2009). 19703397 10.1016/j.cell.2009.05.049 PMC2758036

[16] Martens S., Kozlov M.M., & McMahon H.T. How synaptotagmin promotes membrane fusion. Science. 316, 1205–1208 (2007). 17478680 10.1126/science.1142614

[17] Wu Z. & Schulten K. Synaptotagmin’s role in neurotransmitter release likely involves Ca(2+)-induced conformational transition. Biophys. J. 107, 1156–1166 (2014). 25185551 10.1016/j.bpj.2014.07.041 PMC4156666

[18] AraÃ§ D. Close membrane-membrane proximity induced by Ca(2+)-dependent multivalent binding of synaptotagmin-1 to phospholipids. Nat. Struct. Mol. Biol. 13, 209–217 (2006). 16491093 10.1038/nsmb1056

[19] Ma L. Single-molecule force spectroscopy of protein-membrane interactions. Elife. 6. pii: e30493. doi: 10.7554/eLife.30493., e30493 (2017). 29083305 PMC5690283

[20] van den Bogaart G. Synaptotagmin-1 may be a distance regulator acting upstream of SNARE nucleation. Nat. Struct. Mol. Biol. 18, 805–812 (2011). 21642968 10.1038/nsmb.2061 PMC3130798

[21] Zhou Q. The primed SNARE-complexin-synaptotagmin complex for neuronal exocytosis. Nature. 548, 420–425 (2017). 28813412 10.1038/nature23484 PMC5757840

[22] Zhou Q. Architecture of the synaptotagmin-SNARE machinery for neuronal exocytosis. Nature 525, 62–67 (2015). 26280336 10.1038/nature14975 PMC4607316

[23] Lai Y. Inhibition of calcium-triggered secretion by hydrocarbon-stapled peptides. Nature 603, 949–956 (2022). 35322233 10.1038/s41586-022-04543-1 PMC8967716

[24] Grushin K. Structural basis for the clamping and Ca(2+) activation of SNARE-mediated fusion by synaptotagmin. Nat. Commun. 10, 2413–10391 (2019). 31160571 10.1038/s41467-019-10391-x PMC6546687

[25] Brewer K.D. Dynamic binding mode of a Synaptotagmin-1-SNARE complex in solution. Nat. Struct. Mol. Biol. 22, 555–564 (2015). 26030874 10.1038/nsmb.3035 PMC4496268

[26] Mendonca P.R.F. Asynchronous glutamate release is enhanced in low release efficacy synapses and dispersed across the active zone. Nat. Commun. 13, 3497 (2022). 35715404 10.1038/s41467-022-31070-4 PMC9206079

[27] Chamberland S. Slow-decaying presynaptic calcium dynamics gate long-lasting asynchronous release at the hippocampal mossy fiber to CA3 pyramidal cell synapse. Synapse. 74, e22178 (2020). 32598500 10.1002/syn.22178 PMC7685170

[28] Chamberland S., Timofeeva Y., Evstratova A., Volynski K., & Toth K. Action potential counting at giant mossy fiber terminals gates information transfer in the hippocampus. Proc. Natl. Acad. Sci. U. S. A. 115, 7434–7439 (2018). 29946034 10.1073/pnas.1720659115 PMC6048548

[29] Timofeeva Y. & Volynski K.E. Calmodulin as a major calcium buffer shaping vesicular release and short-term synaptic plasticity: facilitation through buffer dislocation. Front Cell Neurosci. 9:239. doi: 10.3389/fncel.2015.00239. eCollection@2015., 239 (2015). 26190970 PMC4486835

[30] Ermolyuk Y.S. Differential triggering of spontaneous glutamate release by P/Q-, N- and R-type Ca(2+) channels. Nat. Neurosci. 16, 1754–1763 (2013). 24185424 10.1038/nn.3563 PMC4176737

[31] Sinha R., Ahmed S., Jahn R., & Klingauf J. Two synaptobrevin molecules are sufficient for vesicle fusion in central nervous system synapses. Proc. Natl. Acad. Sci. U. S. A. 108, 14318–14323 (2011). 21844343 10.1073/pnas.1101818108 PMC3161593

[32] Mohrmann R., de W.H., Verhage M., Neher E., & SÃ¸rensen J.B Fast vesicle fusion in living cells requires at least three SNARE complexes. Science 330, 502–505 (2010). 20847232 10.1126/science.1193134

[33] Ramakrishnan S., Bera M., Coleman J., Rothman J.E., & Krishnakumar S.S. Synergistic roles of Synaptotagmin-1 and complexin in calcium-regulated neuronal exocytosis. Elife. 9:e54506. doi: 10.7554/eLife.54506., e54506 (2020). 32401194 PMC7220375

[34] Radhakrishnan A. Symmetrical arrangement of proteins under release-ready vesicles in presynaptic terminals. Proc. Natl. Acad. Sci. U. S. A. 118, e2024029118 (2021). 33468631 10.1073/pnas.2024029118 PMC7865176

[35] Paddock B.E. Membrane penetration by synaptotagmin is required for coupling calcium binding to vesicle fusion in vivo. J. Neurosci. 31, 2248–2257 (2011). 21307261 10.1523/JNEUROSCI.3153-09.2011 PMC3092483

[36] Rhee J.S. Augmenting neurotransmitter release by enhancing the apparent Ca2+ affinity of synaptotagmin 1. Proc. Natl. Acad. Sci. U. S. A. 102, 18664–18669 (2005). 16352718 10.1073/pnas.0509153102 PMC1311909

[37] Voleti R., Jaczynska K., & Rizo J. Ca(2+)-dependent release of synaptotagmin-1 from the SNARE complex on phosphatidylinositol 4,5-bisphosphate-containing membranes. Elife. 9:e57154. doi: 10.7554/eLife.57154., e57154 (2020). 32808925 PMC7498268

[38] Mackler J.M., Drummond J.A., Loewen C.A., Robinson I.M., & Reist N.E. The C(2)B Ca(2+)-binding motif of synaptotagmin is required for synaptic transmission in vivo. Nature. 418, 340–344 (2002). 12110842 10.1038/nature00846

[39] Xu J., Pang Z.P., Shin O.H., & Sudhof T.C. Synaptotagmin-1 functions as a Ca2+ sensor for spontaneous release. Nat. Neurosci. 12, 759–766 (2009). 19412166 10.1038/nn.2320 PMC2739891

[40] Bai J., Tucker W.C., & Chapman E.R. PIP2 increases the speed of response of synaptotagmin and steers its membrane-penetration activity toward the plasma membrane. Nat. Struct. Mol. Biol. 11, 36–44 (2004). 14718921 10.1038/nsmb709

[41] Perez-Lara A. PtdInsP2 and PtdSer cooperate to trap synaptotagmin-1 to the plasma membrane in the presence of calcium. Elife. 5. pii: e15886. doi: 10.7554/eLife.15886., e15886 (2016). 27791979 PMC5123861

[42] Francois-Martin C., Rothman J.E., & Pincet F. Low energy cost for optimal speed and control of membrane fusion. Proc. Natl. Acad. Sci. U. S. A. 114, 1238–1241 (2017). 28115718 10.1073/pnas.1621309114 PMC5307435

[43] Manca F. SNARE machinery is optimized for ultrafast fusion. Proc. Natl. Acad. Sci. U. S. A 116, 2435–2442 (2019). 30700546 10.1073/pnas.1820394116 PMC6377469

[44] Radhakrishnan A., Stein A., Jahn R., & Fasshauer D. The Ca2+ affinity of synaptotagmin 1 is markedly increased by a specific interaction of its C2B domain with phosphatidylinositol 4,5-bisphosphate. J. Biol. Chem. 284, 25749–25760 (2009). 19632983 10.1074/jbc.M109.042499 PMC2757977

[45] Voleti R., Tomchick D.R., Sudhof T.C., & Rizo J. Exceptionally tight membrane-binding may explain the key role of the synaptotagmin-7 C2A domain in asynchronous neurotransmitter release. Proc. Natl. Acad. Sci. U. S. A. %201710708. doi: 10.1073/pnas.1710708114., 10 (2017). PMC5635908 28923929

[46] Hui E. Three distinct kinetic groupings of the synaptotagmin family: candidate sensors for rapid and delayed exocytosis. Proc. Natl. Acad. Sci. U. S. A. 102, 5210–5214 (2005). 15793006 10.1073/pnas.0500941102 PMC556003

[47] Brandt D.S., Coffman M.D., Falke J.J., & Knight J.D. Hydrophobic contributions to the membrane docking of synaptotagmin 7 C2A domain: mechanistic contrast between isoforms 1 and 7. Biochemistry 51, 7654–7664 (2012). 22966849 10.1021/bi3007115 PMC3494482

[48] Davis A.F. Kinetics of synaptotagmin responses to Ca2+ and assembly with the core SNARE complex onto membranes. Neuron. 24, 363–376 (1999). 10571230 10.1016/s0896-6273(00)80850-8

[49] Gillespie D.T. Exact stochastic simulation of coupled chemical reactions. J. Phys. Chem. 81, 2340–2361 (1977).

[50] Schneggenburger R. & Neher E. Intracellular calcium dependence of transmitter release rates at a fast central synapse. Nature 406, 889–893 (2000). 10972290 10.1038/35022702

[51] Bollmann J.H., Sakmann B., & Borst J.G. Calcium sensitivity of glutamate release in a calyx-type terminal. Science. 289, 953–957 (2000). 10937999 10.1126/science.289.5481.953

[52] Lou X., Scheuss V., & Schneggenburger R. Allosteric modulation of the presynaptic Ca2+ sensor for vesicle fusion. Nature. 435, 497–501 (2005). 15917809 10.1038/nature03568

[53] Sun J. A dual-Ca2+-sensor model for neurotransmitter release in a central synapse. Nature 450, 676–682 (2007). 18046404 10.1038/nature06308 PMC3536472

[54] Xu J., Mashimo T., & Sudhof T.C. Synaptotagmin-1, −2, and −9: Ca(2+) sensors for fast release that specify distinct presynaptic properties in subsets of neurons. Neuron. 54, 567–581 (2007). 17521570 10.1016/j.neuron.2007.05.004

[55] Bucurenciu I., Kulik A., Schwaller B., Frotscher M., & Jonas P. Nanodomain coupling between Ca2+ channels and Ca2+ sensors promotes fast and efficient transmitter release at a cortical GABAergic synapse. Neuron. 57, 536–545 (2008). 18304483 10.1016/j.neuron.2007.12.026

[56] Nadkarni S., Bartol T.M., Sejnowski T.J., & Levine H. Modelling vesicular release at hippocampal synapses. PLoS Comput. Biol. 6, e1000983 (2010). 21085682 10.1371/journal.pcbi.1000983 PMC2978677

[57] Zucker R.S. & Regehr W.G. Short-term synaptic plasticity. Annu. Rev. Physiol. 64:355–405., 355–405 (2002). 11826273 10.1146/annurev.physiol.64.092501.114547

[58] Evstratova A. & Toth K. Information processing and synaptic plasticity at hippocampal mossy fiber terminals. Front Cell Neurosci. 8, 28 (2014). 24550783 10.3389/fncel.2014.00028 PMC3912358

[59] Vyleta N.P. & Jonas P. Loose coupling between Ca2+ channels and release sensors at a plastic hippocampal synapse. Science. 343, 665–670 (2014). 24503854 10.1126/science.1244811

[60] Lai Y. Molecular Mechanisms of Synaptic Vesicle Priming by Munc13 and Munc18. Neuron. 95, 591–607 (2017). 28772123 10.1016/j.neuron.2017.07.004 PMC5747255

[61] Jaczynska K. Analysis of tripartite Synaptotagmin-1-SNARE-complexin-1 complexes in solution. FEBS Open. Bio(2022). 10.1002/2211-5463.13503 PMC9811660 36305864

[62] Brunger A.T. & Leitz J. The core complex of the Ca(2+)-triggered presynaptic fusion machinery. J. Mol. Biol. 167853 (2022). 36243149 10.1016/j.jmb.2022.167853 PMC10578080

[63] Raingo J. VAMP4 directs synaptic vesicles to a pool that selectively maintains asynchronous neurotransmission. Nat. Neurosci. 15, 738–745 (2012). 22406549 10.1038/nn.3067 PMC3337975

[64] Scheuber A. Loss of AP-3 function affects spontaneous and evoked release at hippocampal mossy fiber synapses. Proc. Natl. Acad. Sci. U. S. A 103, 16562–16567 (2006). 17056716 10.1073/pnas.0603511103 PMC1637621

[65] Yao J., Gaffaney J.D., Kwon S.E., & Chapman E.R. Doc2 is a Ca2+ sensor required for asynchronous neurotransmitter release. Cell. 147, 666–677 (2011). 22036572 10.1016/j.cell.2011.09.046 PMC3220409

[66] Wu Z. Polybasic Patches in Both C2 Domains of Synaptotagmin-1 Are Required for Evoked Neurotransmitter Release. J. Neurosci. 42, 5816–5829 (2022). 35701163 10.1523/JNEUROSCI.1385-21.2022 PMC9337609

[67] Tagliatti E. Synaptotagmin 1 oligomers clamp and regulate different modes of neurotransmitter release. Proc. Natl. Acad. Sci. U. S. A. 117, 3819–3827 (2020). 32015138 10.1073/pnas.1920403117 PMC7035618

[68] Wang J. Calcium sensitive ring-like oligomers formed by synaptotagmin. Proc. Natl. Acad. Sci. U. S. A. 201415849 (2014). 10.1073/pnas.1415849111 PMC4183308 25201968

[69] Eggermann E., Bucurenciu I., Goswami S.P., & Jonas P. Nanodomain coupling between Ca(2+) channels and sensors of exocytosis at fast mammalian synapses. Nat. Rev. Neurosci. 13, 7–21 (2011). 22183436 10.1038/nrn3125 PMC3617475

[70] Naraghi M. T-jump study of calcium binding kinetics of calcium chelators. Cell Calcium. 22, 255–268 (1997). 9481476 10.1016/s0143-4160(97)90064-6

[71] Nagerl U.V., Novo D., Mody I., & Vergara J.L. Binding kinetics of calbindin-D(28k) determined by flash photolysis of caged Ca(2+). Biophys. J. 79, 3009–3018 (2000). 11106608 10.1016/S0006-3495(00)76537-4 PMC1301179

[72] Goswami S.P., Bucurenciu I., & Jonas P. Miniature IPSCs in Hippocampal Granule Cells Are Triggered by Voltage-Gated Ca2+ Channels via Microdomain Coupling. J. Neurosci. 32, 14294–14304 (2012). 23055500 10.1523/JNEUROSCI.6104-11.2012 PMC3632771

[73] Meinrenken C.J., Borst J.G., & Sakmann B. Calcium secretion coupling at calyx of held governed by nonuniform channel-vesicle topography. J. Neurosci. 22, 1648–1667 (2002). 11880495 10.1523/JNEUROSCI.22-05-01648.2002 PMC6758886

[74] Faas G.C., Raghavachari S., Lisman J.E., & Mody I. Calmodulin as a direct detector of Ca2+ signals. Nat. Neurosci. 14, 301–304 (2011). 21258328 10.1038/nn.2746 PMC3057387

[75] Xia Z. & Storm D.R. The role of calmodulin as a signal integrator for synaptic plasticity. Nat. Rev. Neurosci. 6, 267–276 (2005). 15803158 10.1038/nrn1647

