# Supplementary material for: The release of inhibition model reproduces kinetics and plasticity of neurotransmitter release in central synapses
Source: Res Sq. 2023 Apr 27:rs.3.rs-2700789. Preprint. [Version 1] doi: 10.21203/rs.3.rs-2700789/v1 (PMC10168444; doi:10.21203/rs.3.rs-2700789/v1)
Supplement: Supplement 1 [file NIHPPrs2700789v1-supplement-1.pdf]

Supplementary Information

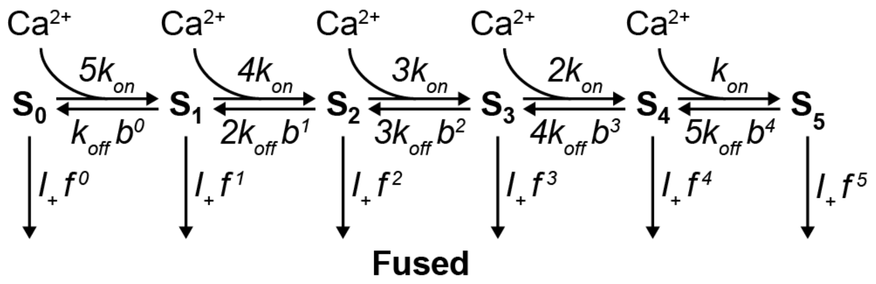

Supplementary Figure 1. Benchmark allosteric model

Six-state allosteric model of Ca^{2+} activation of vesicle fusion in the calyx of Held described in ref. [52]. The model parameters are: k_{on} = 0.1 μM^{-1} ms^{-1}, k_{off} = 4 ms^{-1}, b = 0.5, f = 31.3, and l_+ = 2 × 10^{-7} ms^{-1}. The model was stochastically simulated identically to the release of inhibition models (see Methods).

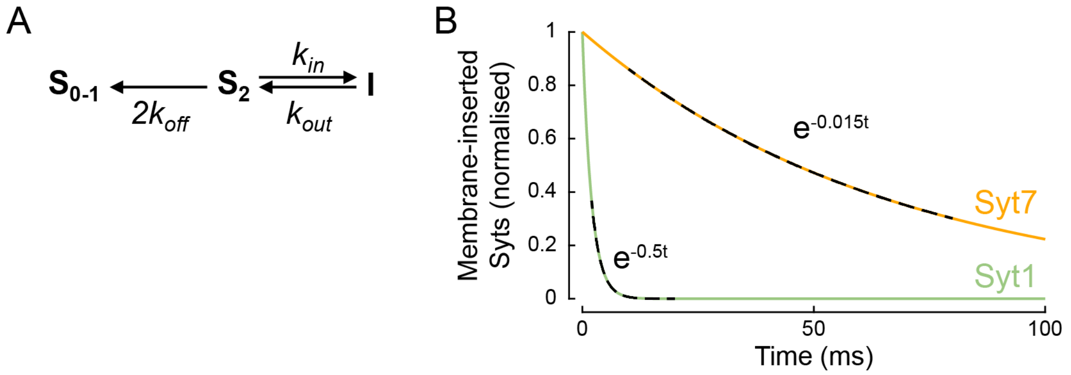

Supplementary Figure 2. Estimation of Syt1 and Syt7 C2 domain membrane dissociation rates from stopped-flow experimental data.

(A) Reduced kinetic scheme describing synaptotagmin C2 domain interaction with a lipid bilayer in a stopped-flow experiment. When Ca^{2+} is removed from the system the lowest states in the kinetic model (Figure 1B), states S_0 and S_1, can be combined into a single absorbing state S_{0-1}. The dynamics of the non-absorbing states, S_2 and I, are described by the system of first order ODEs:

$$\frac{dP(S_2)}{dt} = k_{out}P(I) - (k_{in} + 2k_{off})P(S_2)$$

$$\frac{dP(I)}{dt} = k_{in}P(S_2) - k_{out}P(I).$$

The general solution for this system can be expressed as $P(I) = Ae^{-\lambda_1 t} + Be^{-\lambda_2 t}$, where A and B are constants and the rate parameters are given by $\lambda_{1,2} = \frac{-(k_{out} + 2k_{off} + k_{in}) \pm \sqrt{(k_{out} + 2k_{off} + k_{in})^2 - 8k_{off}k_{out}}}{2}$. From the solutions for $\lambda_{1,2}$, using the previously constrained values of $k_{on} = 1$ µM^{-1} ms^{-1}, $k_{off} = 150$ ms^{-1}, and k_{in} = 100 ms^{-1}, the fast component λ_2 > 400 ms^{-1} for all positive values of k_{out}, and would therefore dissipate well within the dead time of the stopped-flow apparatus (> 1 ms) [47]. This means that the slower exponential component dominates the model dynamics over timescales observed in the stopped-flow experiments, and the simplification $P(I) \propto e^{-\lambda_1 t}$ should provide an appropriate approximation of the experimental data. The full expansion of λ_1 can then be equated with the apparent membrane dissociation rate of Syt1 or Syt7, k_{diss}, to complete the system of kinetic parameters with: $k_{out} = k_{diss}\left(1 - \frac{k_{in}}{k_{diss} - 2k_{off}}\right)$. For the value of k_{diss} = 0.5 ms^{-1} this equation yields k_{out} = 0.67 ms^{-1} for Syt1, and for k_{diss} = 0.015 ms^{-1} it yields k_{out} = 0.02 ms^{-1} for Syt7.

(B) Simulated proportion of membrane-inserted Syt1 and Syt7 when Ca^{2+} is instantaneously removed from the system (i.e. after fast dilution in EGTA containing buffer). The membrane dissociation curves closely follow single exponential decay functions with the rate constants from stopped-flow experiments, indicating a negligible contribution from the secondary minor exponential component.

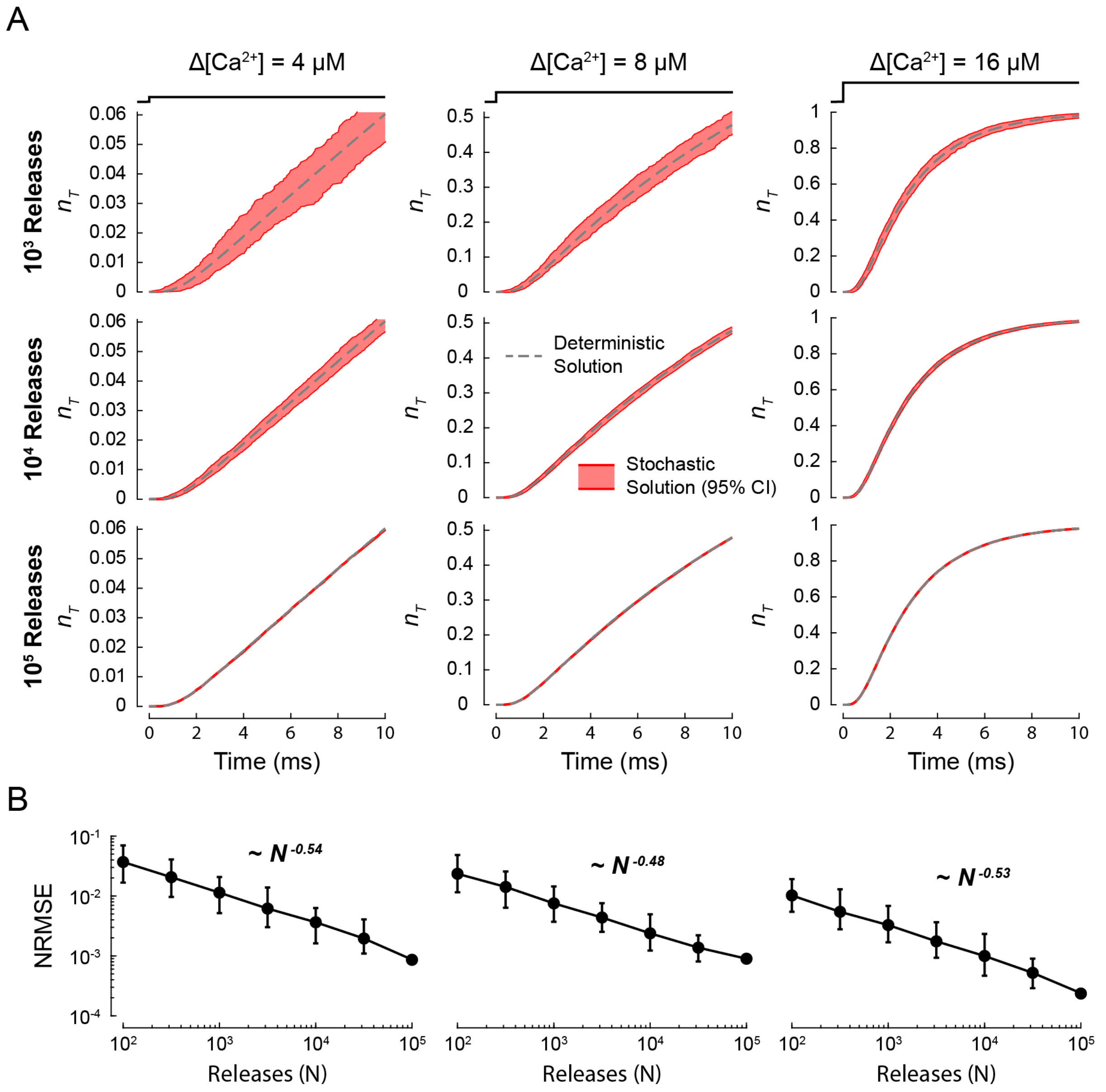

Supplementary Figure 3. Convergence of Monte Carlo estimates to exact solution with the allosteric model.

Stochastic simulations of the allosteric model were performed in response to 100 ms [Ca^{2+}] steps at 4, 8, and 16 μM, without vesicle replenishment, until 100,000 release events were achieved. Monte Carlo estimates of $n_T(t)$ were then calculated, as described in Methods, from subsamples drawn from these release event collections and compared against the exact solutions of the differential master equations.

(A) The first 10 ms of simulations in response to each [Ca^{2+}] step (indicated above) for subsample sizes of 1,000, 10,000, and 100,000 release events with 95% confidence intervals calculated from 100 subsamples (shaded areas).

(B)The typical deviation of Monte Carlo estimates with N stochastic simulations from the deterministic solution was quantified as the normalised root mean squared error (NRMSE) which is given by

$$NRMSE = \frac{\sqrt{\frac{1}{N}\sum_{i=1}^{N}\left(Y_i - \hat{Y}_i\right)^2}}{\max_i\left(\hat{Y}_i\right) - \min_i\left(\hat{Y}_i\right)}$$

where Y_i are the Monte Carlo predictions and $\hat{Y}_i$ are the corresponding deterministic values. The NRMSE is shown for release time subsample sizes between 100 and 100,000. Data are presented as mean values with 95% confidence intervals calculated from 100 subsamples. Slopes of best fit over the range are shown, indicating that NRMSE scales with the number of recorded release times, N, roughly according to $\frac{1}{\sqrt{N}}$. When all 100,000 release times are used to generate Monte Carlo predictions the maximal NRMSE is 0.0867%. In the worst-case scenario where only 2,250 release times are recorded, the maximal NRMSE (estimated from linear interpolation between mean values) is 0.898%.

Supplementary Table 1. Properties of Ca^{2+} buffers used in VCell models of presynaptic Ca^{2+} dynamics.

Calbindin-D28K (Supplementary refs.[69-72])

Reactions:

$$CB_{fast} + Ca^{2+} \underset{k_{off}^{CB\_fast}}{\overset{k_{on}^{CB\_fast}}{\rightleftarrows}} CaCB_{fast}$$

$$CB_{slow} + Ca^{2+} \underset{k_{off}^{CB\_slow}}{\overset{k_{on}^{CB\_slow}}{\rightleftarrows}} CaCB_{slow}$$

Reaction rates:

$k_{on}^{CB\_fast}$	8.7 x 10^{-2} μM^{-1} ms^{-1}
$k_{off}^{CB\_fast}$	3.58 x 10^{-2} ms^{-1}
$[CB_{fast}]_{total}$	95 μM
$k_{on}^{CB\_slow}$	1.1 x 10^{-2} μM^{-1} ms^{-1}
$k_{off}^{CB\_slow}$	2.6 x 10^{-2} ms^{-1}
$[CB_{slow}]_{total}$	95 μM
D_{CB}	2 x 10^{-2} μm^2 ms^{-1}

ATP (Supplementary refs.[72-74])

Reactions:

$$ATP + Ca^{2+} \underset{k_{off}^{ATP}}{\overset{k_{on}^{ATP}}{\rightleftarrows}} CaATP$$

Reaction rates:

k_{on}^{ATP}	0.5 μM^{-1} ms^{-1}
k_{off}^{ATP}	100 ms^{-1}
$[ATP]_{total}$	0.9 mM (corresponding to 58 μM $[ATP]_{free}$ at 1 mM $[Mg^{2+}]_{free}$ [74])
D_{ATP}	0.22 μm^2 ms^{-1}

Calmodulin (Supplementary refs.[74, 75])

Reactions:

N-lobe

$$N_TN_T + Ca^{2+} \underset{k_{off}^{(T),N}}{\overset{2\cdot k_{on}^{(T),N}}{\rightleftarrows}} CaN_TN_R + Ca^{2+} \underset{2\cdot k_{off}^{(R),N}}{\overset{k_{on}^{(R),N}}{\rightleftarrows}} CaN_RCaN_R$$

C-lobe

$$C_TC_T + Ca^{2+} \underset{k_{off}^{(T),C}}{\overset{2\cdot k_{on}^{(T),C}}{\rightleftarrows}} CaC_TC_R + Ca^{2+} \underset{2\cdot k_{off}^{(R),C}}{\overset{k_{on}^{(R),C}}{\rightleftarrows}} CaC_RCaC_R$$

Reaction rates:

N-lobe

$k_{on}^{(T),N}$	0.77 μM^{-1} ms^{-1}
$k_{off}^{(T),N}$	160 ms^{-1}
$k_{on}^{(R),N}$	32 μM^{-1} ms^{-1}
$k_{off}^{(R),N}$	22 ms^{-1}

C-lobe

$k_{on}^{(T),C}$	8.4 x 10^{-2} μM^{-1} ms^{-1}

$k_{off}^{(T),C}$	2.6 ms^{-1}
$k_{on}^{(R),C}$	2.5 x 10^{-2} μM^{-1} ms^{-1}
$k_{off}^{(R),C}$	6.5 x 10^{-3} μM^{-1} ms^{-1}
$[CaM]_{total}$	100 μM
D_{CaM}	2 x 10^{-2} μm^2 ms^{-1}

Supplementary Files

This is a list of supplementary files associated with this preprint. Click to download.

- Normanetalcode.zip
